# Thyroid hormones association with depression severity and clinical outcome in patients with major depressive disorder

**DOI:** 10.1007/s11033-014-3097-6

**Published:** 2014-01-18

**Authors:** Dominika Berent, Krzysztof Zboralski, Agata Orzechowska, Piotr Gałecki

**Affiliations:** 1 Department of Adult Psychiatry, Medical University of Lodz, Aleksandrowska 159 Str., 91-229 Lodz, Poland; 2 Babiński Memorial Hospital, Aleksandrowska 159 Str., 91-229 Lodz, Poland

**Keywords:** Major depression, Thyroid hormones, Treatment, Outcome

## Abstract

The clinical implications of thyroid hormones in depression have been studied extensively and still remains disputable. Supplementation of thyroid hormones is considered to augment and accelerate antidepressant treatment. Studies on the role of thyroid hormones in depression deliver contradictory results. Here we assess theirs impact on depression severity and final clinical outcome in patients with major depression. Thyrotropin, free thyroxine (FT4), and free triiodothyronine (FT3) concentrations were measured with automated quantitative enzyme immunoassay. Depression severity and final clinical outcome were rated with 17-itemic Hamilton Rating Scale for Depression [HDRS(17)] and Clinical Global Impression Scales for severity and for improvement (CGIs, CGIi). FT3 and FT4 concentrations were significantly positively correlated with clinical improvement evaluated with CGIi (R = 0.38, *P* = 0.012; R = 0.33, *P* = 0.034, respectively). There was a significant correlation between FT4 concentrations and depression severity assessed in HDRS(17) (R = 0.31, *P* = 0.047). Male patients presented significantly higher FT3 serum levels (Z = 2.34, *P* = 0.018) and significantly greater clinical improvement (Z = 2.36, *P* = 0.018) when compared to female patients. We conclude that free thyroid hormones concentrations are associated with depression severity and have an impact on final clinical outcome. It can be more efficient to augment and accelerate the treatment of major depressive disorder with triiodothyronine instead of levothyroxine because of individual differences in thyroid hormones metabolism.

## Introduction

The clinical implications of thyroid hormones in depression have been studied extensively and still remains disputable. Maes et al. [1] indicated that basal morning thyrotropin (TSH), free triiodothyronine (FT3) and free thyroxine (FT4) plasma levels fell within the normal, euthyroid range in 96.8 % of depressed patients. It is noteworthy that reduction of basal TSH and elevation of FT4, however still not extending normative range, were found to be common in major depressive disorder [2]. Nonetheless Sintzel et al. [3] estimated the prevalence of subclinical hypothyroidism in population of resistant depression and simple depression as: 52 and 8–17 %, respectively. To compare, the prevalence of an overt (elevated TSH levels and low levels of FT3 and FT4) and subclinical (elevated TSH levels only) hypothyroidism in adult population and is 9 and 0.4 %, respectively [4]. It is remarkable that the disturbances in free thyroid hormones levels can be isolated, accompanied by normal TSH level. It can be, i.e., due to deiodinases defects, enzymes converting prohormone FT4 into active FT3 [5]. The measurement of basal TSH, FT3 and FT4 plasma levels was indicated to be more proper than thyrotropin releasing hormone(TRH) assessment for evaluating hypothalamus-pituitary-thyroid axis (HPT axis) function [2].

Thyroid hormones receptors are predominantly present in cerebral cortex, amygdala, plexus choroideus and structures of adult neurogenesis: hipoccampus and olfactory bulb [6]. Thyroid hormones modify expression of genes encoding myelin, neurotrophins, and proteins involved in intracellular signaling pathways [7]. They have also neroprotective and vasodilatory effects [8]. Moreover they induce reduction of the sensitivity of 5-HT1A autoreceptors and increase in 5-HT2 receptor sensitivity [9, 10] which leads to serotonergic neurotransmission elevation.

Thyroid disorders with immune and autoimmune background are accompanied by an increased incidence of depression. The probably reason for this relation is the fact that immune dysregulation is one of known pathophysiological mechanisms underlying the development of depression [11]. Depression is seen in autoimmune thyroid disorders, both in thyroiditis and normal thyroid function [12]. The presence of thyroid peroxidase antibodies was proposed to be a vulnerability marker for depression [13].

The aims of this study were to analyze TSH, FT3 and FT4 measurements in patients referred to our psychiatry ward for a treatment of major depressive disorder (MDD) and to assess levels of TSH, FT3 and FT4 association with depression severity and improvement.

## Materials and methods

### Participants

Data obtained and analyzed in this study were delivered from patients’ archive medical documentation stored in our hospital. The study protocol had been approved by the Local Bioethics Committee No. RNN/126/13/KB.

We enrolled 44 consecutive patients referred to a psychiatric hospital between 2008 and 2013 for a treatment of depression and having their blood samples for TSH, FT3, FT4 obtained during the first week of hospitalization among routine, charged by physician, blood tests. A diagnosis of MDD was established according to the Diagnostic and Statistical Manual of Mental Disorders, Fourth Edition (DSM-IV) diagnostic criteria [14].

Patients with overt (including autoimmune) thyroid disease or already treated for thyroid disease (levothyroxine or antithyroid medication) were excluded. Antidepressant treatment consisted of selective serotonin reuptake inhibitors (SSRIs).

### Hormone assay

The laboratory tests for TSH, FT3 and FT4 were ordered by each patient’s physician among other routine laboratory tests on admission. Morning whole blood samples were centrifugated and TSH, FT3, FT4 serum levels were measured with automated quantitative enzyme immunoassay on the instrument mini Vidas of the BioMerieux Company. A reference values for the local laboratory were for TSH: 0.25–5.00 μIU/ml; FT3: 4.00–8.3 pmol/l; FT4: 10.6–19.40 pmol/l. The inter-assay coefficient of variation (inter-CV) of the assay for TSH concentrations of 33.40 μIU/ml was 2.4 % and the intra-assay coefficient of variation (intra-CV) of the assay for TSH concentrations of 31.40 μIU/ml was 3.2 %. The FT3 assay had the inter-CV of 3.4 % at 12.60 pmol/l and intra-CV of 3.8 % at 12.50 pmol/l. The inter- and intra-CV of the assay for FT4 concentrations of 51.53 pmol/l were 2.3 and 3.8 %, respectively.

### Symptom scales

The severity of depression was rated with the 17-itemic Hamilton Rating Scale for Depression [HRSD(17)] [15, 16] and the Clinical Global Impression Scale for severity (CGIs) [13] at the first week of hospitalization. The improvement was assessed with the Clinical Global Impression Scale for improvement (CGIi) [17] at the last week of hospitalization. All the scales were completed by the same physician.

The highest score in CGIs of 7 points means that patient is among the most extremely ill; a score of 6 indicates that he is severely ill; 5, markedly ill; 4, moderately ill; 3, mildly ill; 2, borderline mentally ill; 1, normal, not at all ill.

The highest score in HRSD(17) is 52 points. Patients scoring between 0 and 7 points have no depression; 8–12 points, slight depression; 13–17 points, moderate depression; 18–29 points, severe depression; 30–52 points, very severe depression.

The highest score in CGIi is 6 points, which indicates ideal improvement; a score of 5 indicates very considerable improvement; 4, considerable improvement; 3, moderate improvement; 2, slight improvement; 1, very slight improvement; 0, state unchanged; −1, very slight deterioration; −2, slight deterioration; −3, moderate deterioration; −4, considerable deterioration; −5, very considerable deterioration; −6, maximum deterioration.

### Data analysis

All data analyses were performed in Statistica (version 10.0). The results were presented as percentages (*%*) or means with standard deviations (±SD). Types of measurements were selected after the analysis of the variables tested, which showed no normal distribution. *P* values less than 0.05 were considered to be significant. We used Mann–Whitney *U*-test to determine differences between current age, age at onset, disease duration, number of hospitalization, number of suicide attempts, rating scores and hormone indices in male and female patients. To evaluate the correlation between free thyroid hormones concentrations and HDRS(17), CGIs and CGIi and the correlation between CGIs and HDRS(17) Spearman’s rank correlation coefficients were estimated.

## Results

### Clinical and demographic characteristic of the study group

The study group consisted of 44 patients (20 women, 24 men) with a mean age of 51.93 ± 11.54 years. Disease duration was about 8.5 ± 8.09 years; age at onset was 43.61 ± 12.66 years; number of hospitalization was 2.58 ± 1.92; number of suicide attempts was 0.67 ± 1.3 (Table 1). No significant differences were found in above listed characteristics between male and female patients (Table 1). Family history of depression was present in 11 patients (25 %); absent in 33 patients (75 %).

**Table 1:** Description of the study group characteristics (*N* = 44) with comparison between male and female patients

		Samples (*n* (%))	Significance M versus F
Sex			
Male		24 (54.5)	
Female		20 (45.4)	
Age			
M ± SD years	51.93 ± 11.54		Z = −1.91, *P* = 0.056
Age at onset	43.61 ± 12.66		Z = 0.08, *P* = 0.934
Mean ± SD years			
Disease duration Mean ± SD years	8.5 ± 8.09		Z = 0.66, *P* = 0.508
Total number of hospitalization Mean ± SD	2.58 ± 1.92		Z = −0.33, *P* = 0.747
Number of suicide attempts Mean ± SD	0.67 ± 1.3		Z = −0.32, *P* = 0.747
Presence of family history of depression		11 (25)	
TSH serum levels		43 (97.73)	
Mean ± SD µIU/ml	1.38 ± 0.99	(24 M, 19F)	
FT4 serum levels		42 (95.45)	
Mean ± SD pmol/l	11.43 ± 2.69	(23 M, 19F)	Z = 0.93, *P* = 0.350
FT3 serum levels		43 (97.73)	
Mean ± SD pmol/l	4.45 ± 0.81	(24 M, 19F)	Z = 2.34, *P* = 0.018
HDRS(17) Mean ± SD range	21.50 ± 7.31 points	44 (100)	Z = −0.67, *P* = 0.502
	8–35 points		
CGIs	4.18 ± 1.02 points	44 (100)	Z = −1.53, *P* = 0.125
Mean ± SD range	2–6 points		
CGIi	4.50 ± 1.05 points		
Mean ± SD range	0–6 points	44 (100)	Z = 2.36, *P* = 0.018

*SD* standard deviation, *Z* Mann-Whitney *U*-test, *P* level of statistical significance, *M* male patients, *F* female patients, *TSH* thyrotropin, *FT4* free thyroxine, *FT3* free triidothyronine, *HDRS(17)* 17-itemic Hamilton Rating Scale for Depression, *CGIs* Clinical Global Impression Scale for severity, *CGIi* Clinical Global Impression Scale for improvement

### Hormone assay results

TSH serum levels were measured in 43 individuals (97.73 %, 24 males, 19 females). One measurement was missed in one woman. All the results were within the normal range (reference values: 0.25–5.00 μIU/ml). The mean value (±SD) was 1.38 (±0.99) μIU/ml (Table 1).

**Fig. 1:**
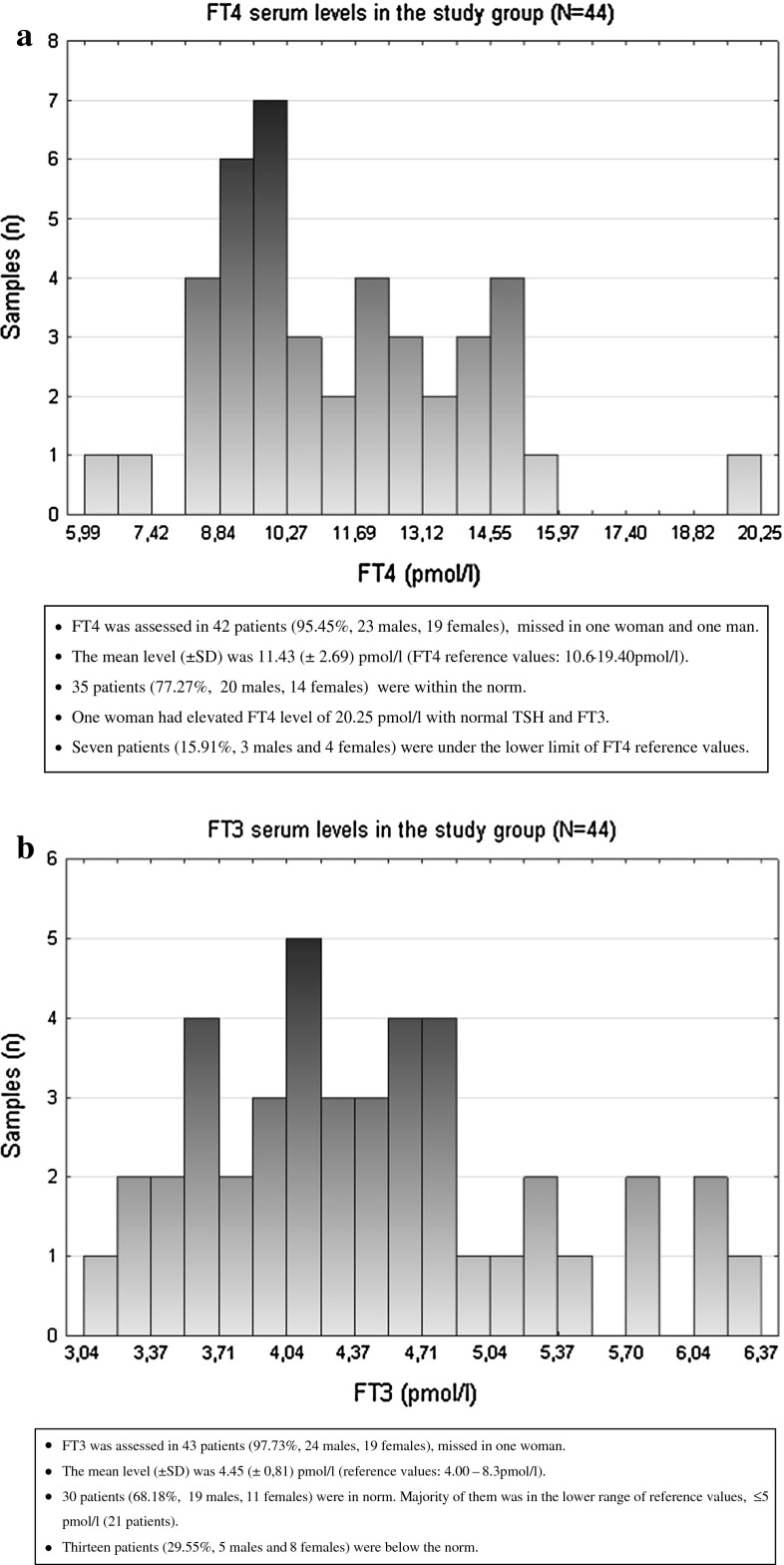
**a** FT4 concentrations in the study group. **b** FT3 concentrations in the study group

FT4 serum concentrations were measured in 42 patients (95.45 %, 23 males, 19 females) (Fig. 1a). They were missed in one woman and one man. The mean level (±SD) was 11.43 (±2.69) pmol/l (FT4 reference values: 10.6–19.40 pmol/l). One woman had elevated FT4 level of 20.25 pmol/l with normal TSH and FT3. Seven patients (15.91 %, three males and four females) were under the lower limit of FT4 reference values. Thirty-five patients (77.27 %, 20 males, 14 females) were within the norm. Further analysis stratified by sex showed no significant difference between FT4 serum levels in male and female patients (Z = 0.93, *P* = 0.350) (Table 1).

FT3 was assessed in 43 patients (97.73 %, 24 males, 19 females), missed in one woman. The mean value (±SD) was 4.45 (±0.81) pmol/l (reference values: 4.00–8.3 pmol/l). 13 patients (29.55 %, five males and eight females) were below the norm; 30 patients (68.18 %, 19 males, 11 females) were in norm. It was noticed that among 30 patients having theirs FT3 within the norm, majority was in the lower range of reference values, ≤5 pmol/l (21 patients) (Fig. 1b). Among patients having FT3 levels within the norm 4 had FT4 below the lower limit of normative range, one had FT4 over the norm and 24 FT4 samples were in norm. Among patients having FT3 below the norm, three had FT4 also below the lower limit of normative range and ten were within the norm. Further analysis stratified by sex showed significant difference between FT3 serum levels in male and female patients (Z = 2.34, *P* = 0.018) and they were greater in men (Table 1).

### Symptom scales rating results

Depression severity was evaluated in all patients (*N* = 44) on admission with CGIs and HDRS(17). Scoring in HDRS(17) reached 21.50 (±7.31) points, range 8–35 (patients with slight to very severe depression). The mean CGIs score (±SD) was 4.18 (±1.02) points, range 2–6 (from patients borderline mentally ill to severely ill). Male and female patients did not differ regarding to rating scores on HDRS(17) and CGIs (Z = −0.67, *P* = 0.502; Z = −1.53, *P* = 0.125, respectively) (Table 1).

CGIi was completed in all patients (*N* = 44) during the last week of hospitalization and the mean value (±SD) was 4.50 (±1.05) points, range 0–6 (from patients with unchanged state to patients with ideal improvement). Male patients improved significantly more than female patients (Z = 2.36, *P* = 0.018) (Table 1).

We assessed whether the results obtained in two scales for depression severity were positively associated and we confirmed significant direct correlation between HDRS(17) and CGIs (R = 0.58, *P* = 0.00003) which, in our opinion, approved correctness of both scales sampling.

### Association between hormone levels and symptom scales rating

FT3 serum levels on admission were positively correlated with clinical improvement evaluated with CGIi and it was significant (R = 0.38, *P* = 0.012) and negatively–with depression severity assessed with CGIs but it was not significant (R = −0.16, *P* = 0.318). There was no association between FT3 concentrations and evaluation with HDRS(17) (R = 0.08, *P* = 0.614). We can assume that lower FT3 on admission was seen in patients with more severe depression which was not significant and was predictive of significantly worse clinical improvement (Table 2).

**Table 2:** Correlation between FT3 (*n* = 43) and FT4 (*n* = 42) serum levels in the study group (*N* = 44) and clinical evaluation with HDRS(17), CGIs, CGIi

	HDRS(17)	CGIs	CGIi
FT3 (pmol/l)	R = 0.08	R = −0.16	R = 0.38
*n* = 43 (24 M, 19F)	*P* = 0.614	*P* = 0.318	*P* = 0.012
FT4 (pmol/l)	R = 0.31	R = 0.16	R = 0.33
*n* = 42 (23 M, 19 F)	*P* = 0.047	*P* = 0.304	*P* = 0.034

*R* Spearman’s rank correlation coefficient, *P* level of statistical significance, *FT4* free thyroxine concentrations, *FT3* free triiodothyronine concentrations, *HDRS(17)* 17-itemic Hamilton Rating Scale for Depression, *CGIs* Clinical Global Impression Scale for severity, *CGIi* Clinical Global Impression Scale for improvement, *M* male patients, *F* female patients

There was a positive, significant association between FT4 concentrations and depression severity assessed in HDRS(17) (R = 0.31, *P* = 0.047), positive but not significant correlation between FT4 concentrations and depression severity evaluated with CGIs (R = 0.16, *P* = 0.304). FT4 serum levels were positively and significantly associated with CGIi (R = 0.33, *P* = 0.034). Here we can conclude that in our population higher FT4 on admission was significantly associated with more severe depression and greater improvement (Table 2).

## Discussion

Our MDD patients were found to display disturbances in free thyroid hormones levels and to have TSH levels within the normative range. Lower FT3 and FT4 concentrations on admission were significant predictors of worse clinical improvement. Moreover male patients presented significantly higher FT3 levels and better clinical improvement. We hypothesize that sex related differences in FT3 concentrations may be connected with different free thyroid hormone metabolism which requires further investigation. Taking into consideration above findings, we suspect triiodothyronine instead of levothyroxine supplementation, more efficient in augmentation and acceleration of the treatment of depression.

The prevalence of thyroid disorders rises with age. They become common in individuals aged 60 years and older [18]. In our population mean age (±SD) was of 51.93 (±11.54) years. TSH level was between reference values in each individual, so there was no apparent, so far undiagnosed, thyroid disorder. However we found FT3 level diminished in 13 patients (29.55 %, five men and eight women) and in norm in 30 patients (68.18 %, 19 males, 11 females). It is worth to add that among patients with normative FT3 ranges, majority (21 patients) presented with ≤5 pmol/l FT3 concentrations. There were also disturbances in FT4 serum levels: seven patients (15.91 %, three men and four women) were below the FT4 norm, 35 patients (77,27 %, 20 males, 14 females) were within the norm and one female presented FT4 above the reference upper limit. Overt thyroid disease was one of exclusion criteria in our study. Thus all TSH measures were within normative ranges. It is noteworthy that Ordas and Labatte (1995) [19] found overt thyroid disease rare among depressed inpatients. They reviewed thyroid function tests obtained on 277 consecutive first time admitted patients with major depression or dysthymia and found TSH outside the normal range in 17 patients (6.5 %). Of these, there were two cases (0.4 %) suggestive of hyperthyroidism and no overt cases of hypothyroidism. Eight patients had subclinical hypothyroidism (elevated TSH, normal T4). They concluded that thyroid screening may add little to diagnostic evaluation in depressed inpatients. However further studies on depressed population, including ours, describe slight free thyroid hormones disturbances with concomitant normal TSH. Moreover we indicate a possible correlation between free thyroid hormone levels and severity and improvement in MDD patients [19].

We mentioned that autoimmune thyroid disorders are frequently accompanied by depression [11]. We have excluded all the patients with overt, previously diagnosed thyroid disorders, including autoimmune disorders. The study group do not contain patients with grossly elevated, indicating thyroid disorder, antibodies. Many depressed patients display slight presence of thyroid antibodies but the clinical meaning of this finding remains unclear because it is predominantly accompanied by normal TSH [20]. In our opinion it can be a part of immune dysregulation in depression but it was not the aim of this study to assess theirs level and possible impact on depression severity and clinical improvement.

Here we proceed hormone immunoassay in serum. However it is noteworthy that HPT axis function assessment is available both with blood and urine tests. The usefulness of testing the thyroid hormone excretion in 24 h urine remains questionable. Wiersinga et al. [21] described two women with a diagnosis of hypothyroidism based on urine test which was further excluded after the measurement of thyroid hormones blood levels. Li et al. [22] found monitoring urinary iodine but not thyroid hormones useful in pregnant women.

We used HDRS(17) and CGIs scales for depression severity evaluation on admission and CGIi for improvement evaluation at the last week of hospitalization. Although HDRS(17) rating scale is approved operationalisable tool for depression severity evaluation, CGI scales appear to carry a risk of clinician’s judgment bias. Khan et al. (2002) [23] performed a retrospective chart review on the records of 208 depressed adult patients and concluded that HDRS(17) rating scale and Clinical Impressions Rating Scale had similar effect sizes regardless of the type of antidepressant evaluated. In our study all the scales were completed by same physician. We confirmed positive and significant correlation between HDRS(17) and CGIs scoring in our study group which, in our opinion, approved correctness of both scales sampling.

Thyroid hormones play an important role in brain development, and then in the adulthood, they influence structure, perfusion and function of the central nervous system [1]. The mechanisms of thyroid hormones action in the brain cells are complicated, warranted by availability of free hormone, activity of thyroid hormone transporters and receptors, activity of deiodinases [24]. Kirkegaard and Faber (1998) [25] reviewed that in depressed patients FT3 levels were reduced while FT4 levels were elevated. In our opinion it may indicate that low serum levels of active hormone with concomitant good availability of prohormone may be due to altered prohormone metabolism, i.e. deiodinase low activity. To our best knowledge there are only few studies evaluating individual disturbances in deiodinase activity in mood disorders [5, 26, 27]. In our study group male patients did not differ from female patients regarding FT4 concentrations but they presented significantly higher FT3 serum levels and improved significantly better than women via the course of hospitalization. There were no significant differences between male and female patients regarding basic clinical characteristics (current age, age at onset, disease duration, number of hospitalization, number of suicide attempts). We hypothesized the gender-related differences in free thyroid hormones levels may be due to more efficient FT4 into FT3 conversion in males. In our opinion it is hard to assess to which extend it influenced current FT4 concentrations. FT3 is an active form of hormone, displaying its biological function in tissues. We think that if we do not particularly assess thyroid hormone metabolism it is more suitable to take conclusions from FT3 not FT4 correlations with rating scores in general practice. In summary we state that lower FT3 concentrations on admission were not significantly connected with more severe depression and were predictive of significantly worse clinical improvement.

Higher FT4 concentrations on admission were significantly connected with more severe depression and greater clinical improvement. Differences in thyroid hormone metabolism between male and female patients and low sample number could confound our results. There was four male patients more than female patients having theirs FT4 measured and five male patients more than female patients having theirs FT3 measured. We can suspect that higher FT4 concentrations on admission results indirectly in greater improvement because they are a source of active FT3. However this source is used more effectively in males due to more efficient FT4 to FT3 conversion. Probably, if we had few female patients more than male patients, high or even low FT4 concentrations on admission would be connected with poor clinical improvement due to less efficient FT4 to FT3 conversion.

Maes et al. [1] assessed HPT axis function in heterogenic study group of patients with major depression episode, major depressive disorder, melancholic depression and dysthymia. In contrast, our study group comprised patients with major depressive disorder. They found no significant gender- or age-related differences in TSH and thyroid hormones levels. Basal TSH values were significantly lower in melancholic patients than in healthy controls, minor and simple major depressed patients, and in major versus minor depressed subjects. FT4 levels were significantly higher in melancholic patients than in all other subjects. They also found that basal TSH and FT4 levels were significantly correlated with the severity of illness. Tsuru et al. [28] delivered interesting data on HPT axis dysregulation during the major depression episode. They recruited 25 patients (16 women and nine men, 48.1 ± 11.4 years of age, range 22–84) with MDD. Patients who recurred within ten years after remission exhibited significantly higher TSH responses to TRH at the time of admission when compared to those who did not recur. In our opinion, it may suggest that the free thyroid hormone decline at the time of depression recur which makes pituitary gland more vulnerable to TRH stimulation.

All the studied patients were treated with SSRIs so there were no possible difference in influencing final clinical outcome between different antidepressants group. Many studies suggest the role of thyroid hormones supplementation in acceleration and augmentation of antidepressant treatment with SSRIs and tricyclic antidepressants [29, 30]. Cooper-Kazaz et al. (2007) [30] showed that depressed patients with low FT3 on admission benefit more from triiodothyronine supplementation. In our opinion it can be more efficient to augment and accelerate the treatment of major depressive disorder with triiodothyronine instead of levothyroxine because of individual differences in thyroid hormones metabolism efficacy. However it should be done carefully in elderly patients. Gussekloo et al. (2004) [31] found in individuals aged 85 years and older that subclinical hypothyroidism may be related to prolonged survival. The treatment with SSRIs in our study was not supported by thyroid hormones supplementation. Moreover it was showed that SSRIs do not influence thyroid hormone levels to extend the normative ranges [32].

The screening test for thyroid disorders is TSH serum blood test. Further analysis of free thyroid hormones is conducted in case of TSH disturbances. In our opinion in patients with major depressive disorder, routine assessment of all three: TSH, FT3 and FT4 on admission should be disseminated into routine practice settings, first to exclude overt or subclinical thyroid disorder and second, in case of normal TSH to exclude disturbances in free thyroid hormones, which can, how it was confirmed in our study, influence clinical outcome.

## Abbreviations

TSH: Thyrothropin
TRH: Thyrotropin-releasing hormone
FT3: Free triiodothyronine
FT4: Free thyroxine
CGIs: Clinical Global Impression Scale for severity
CGIi: Clinical Global Impression Scale for improvement
HPT axis: Hypothalamus-pituitary-thyroid axis
HRSD(17): 17-itemic Hamilton Rating Scale for Depression
SSRIs: Selective serotonin reuptake inhibitors
Z: Mann–Whitney *U*-test
R: Spearman’s rank correlation coefficient
SD: Standard deviation
P: Level of statistical significance
M: Male patients
F: Female patients
